# SARS CoV-2 infection as a risk factor of preeclampsia and pre-term birth. An interplay between viral infection, pregnancy-specific immune shift and endothelial dysfunction may lead to negative pregnancy outcomes

**DOI:** 10.1080/07853890.2023.2197289

**Published:** 2023-04-19

**Authors:** Aleksander Celewicz, Marta Celewicz, Michał Michalczyk, Paula Woźniakowska-Gondek, Kamila Krejczy, Marcin Misiek, Rafał Rzepka

**Affiliations:** a Department of Gynecology and Obstetrics, Collegium Medicum, University of Zielona Góra, 65-417Zielona Góra, Poland; b Holy Cross Cancer Center Clinical Gynecology, Kielce, Poland

**Keywords:** COVID-19, pregnancy, pre-term birth, preeclampsia, subcellular changes, SARS-CoV-2

## Abstract

**Background:**

Since SARS-CoV-2 (severe acute respiratory syndrome coronavirus 2) was first identified as the cause of Coronavirus disease 19 (COVID-19) it has caused over 649,147,421 infections and over 6,730,382 deaths worldwide. SARS-CoV-2 presents higher infectivity than other coronaviridae (MERS-CoV and SARS-CoV). Pregnant patients, according to previous studies are at high risk of severe COVID-19 course and negative pregnancy outcomes (pre-term birth, low birth weight, preeclampsia, operative delivery and ICU admission with need for mechanical ventilation).

**Methods:**

In this review we focus on the pathophysiology of subcellular changes in COVID-19 and try bring to light the aspects that occur in physiological pregnancy that may cause higher risk of SARS-CoV-2 infection and severe COVID-19 course.

**Results:**

Knowledge of potential interplay between viral infection and physiological changes in pregnancy may point us in the direction of future prophylaxis and treatment in this special population.

**Key Messages**
- SARS-CoV-2 having affinity to ACE-2 and causing it’s downregulation receptor may cause endothelial injury leading to compliment activation and formation of NETs, together with RAS dysregulation this may cause preeclampsia to develop in pregnant patients.
- PTB may occur in patients as an effect of SARS-CoV-2 infection in first or second trimester as an effect of TLR4 pathway dysregulation with lower levels of IFNβ.

## Introduction

Severe acute respiratory syndrome coronavirus 2 (SARS-CoV-2) was identified as the causative agent behind Coronavirus disease 19 (COVID-19) in late 2019. Since then it has already caused over 649,147,421 infections [1] and over 6,730,382 deaths [1] worldwide. In 11 March 2020 WHO declared a state of pandemic. SARS-CoV-2 is an RNA virus related to severe acute respiratory syndrome coronavirus (SARS-CoV) and Middle East respiratory syndrome coronavirus (MERS-CoV) [2]. The whole family of coronaviridae has strong affinity to angiotensin II converting enzyme (ACE2) receptor and can replicate in cells with expression of aforementioned receptor [3,4]. The SARS-CoV-2 pandemic with its high number of asymptomatic and low-symptomatic patients is highly infectious and has claimed a higher death toll than SARS-CoV and MERS-CoV epidemics [5]. Pregnant women with COVID-19 are at higher risk of severe course and negative pregnancy outcomes (caesarean delivery, pre-term birth (PTB), low birth weight, preeclampsia (PE), ICU admission and need for mechanical ventilation). As well as are at higher risk of developing pregnancy specific disorders (PE, PTB and premature preterm rupture of membranes). We focus on the subcellular changes that occur in pregnant women with COVID-19 to try to elucidate how those disorders may be triggered by the infection with SARS-CoV-2.

## Outcomes of COVID-19 in pregnancy

### Microvascular changes in COVID-19 AND PE

#### PE and renin-angiotensin system (RAS)

PE is a disorder specific to pregnancy, complicating 2%–8% of pregnancies worldwide and is defined by The International Society for the Study of Hypertension in Pregnancy as a combination of arterial hypertension and proteinuria (>300 mg/day) present after 20 weeks of gestation, or as arterial hypertension which is accompanied by one of the following: thrombocytopenia, impaired liver function, renal insufficiency, pulmonary oedema, neurological abnormalities, foetal growth restriction (FGR) or placental insufficiency [6–8]. A shift in the balance between Ang II/Ang-(1-7) with the former being boosted and latter presenting declining levels is also present in PE [9,10]. Similar effect is observed in COVID-19, when SARS-CoV-2 binds to ACE-2 and causes it’s downregulation accelerating RAS dysregulation. In effect it may start the cascade of changes that develop into late-onset PE in the III trimester [11].

#### Interplay between endothelial injury and compliment activation

Another key feature of PE is multiorgan endothelial cell dysfunction leading to the most severe forms such as HELLP or eclampsia [12]. Endothelial dysfunction leading to dysregulation of coagulation is also one of the most important mechanisms in severe COVID-19 [11]. As ACE-2 is expressed in endothelial cells it is possible that during severe COVID-19 these cells become infected by SARS-CoV-2 [13,14] leading to immune mediated endothelial injury [15,16]. Resulting endothelitis causes compliment activation and formation of C5b-9 complex in high quantities which causes further activation of the common coagulation pathway, with formation of fibrin deposits and increase in D-Dimer levels [17,18]. Compliment activation is followed by attraction of neutrophils to the loci of endothelial injury, proven by the observation that neutrophilia and specifically high neutrophil-to-lymphocyte ratio (NLR) is a marker of poor prognosis in COVID-19 [19,20].

#### Factors contributing to higher risk of thrombotic microangiopathy (TMA)

**Figure 1.**
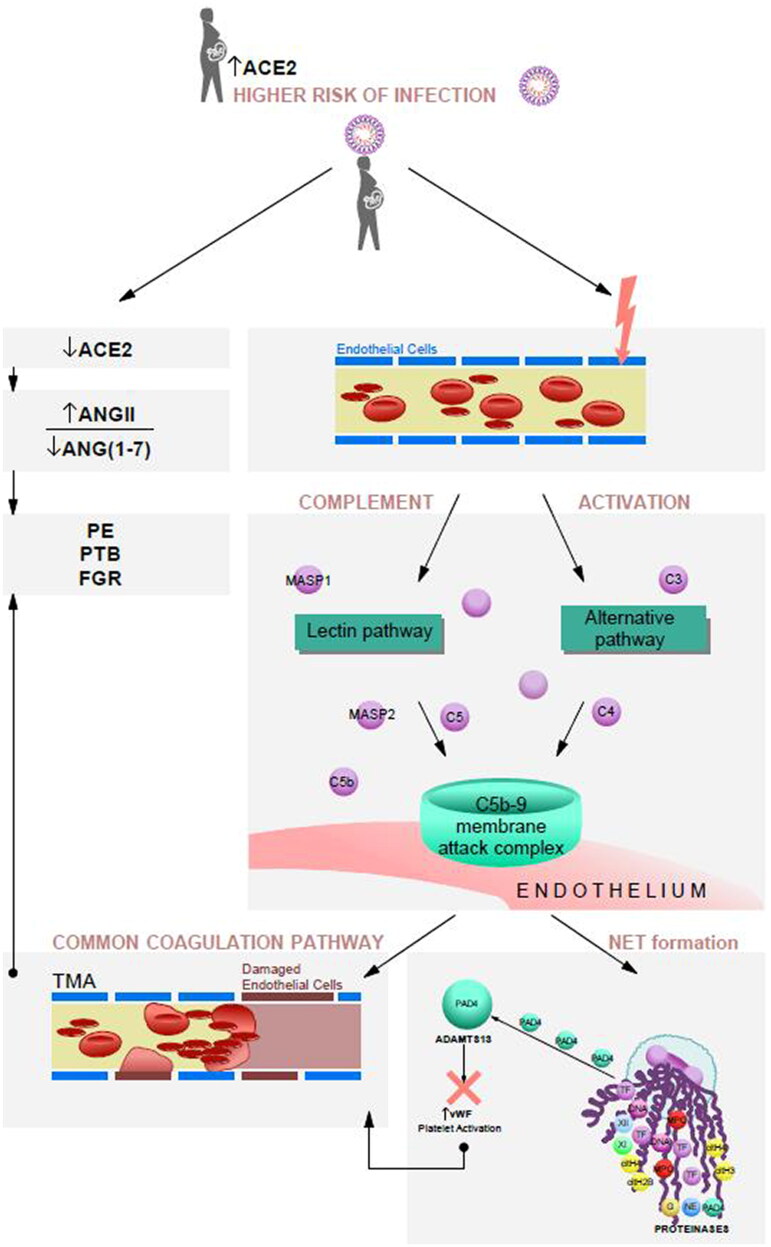
Effect of SARS-CoV-2 infection on risk of PE, PTB and FGR.

Damage-associated molecular patterns are the triggers for recognition receptors sites (Toll-like receptors (TLR) and inflammasomes) binding to neutrophil surface through L-selectin and P-selectin glycoprotein ligand 1 [21]. After activation they undergo degranulation and phagocytosis of the infectious factors may begin, as this process is physiological it should not lead to further cell injury [22]. What may induce TMA is formation of neutrophil extracellular traps (NETs) [23]. This process is composed of decondensation and expansion of chromatin and release of granules to the space surrounding neutrophils [24]. NETs express presence of peptidylearginine deiminase (PAD4) an enzyme that may change a disintegrins and metalloproteinase with thrombospondin type 1 repeats, member 13 (ADAMTS13) structure reducing it’s potential to destroy von Willebrand factor multimers, promoting activation of platelets [25,26]. Usually ADAMTS13 activity is not decreased during acute phase of inflammation but majority of patients with COVID-19 were presenting lower ADAMTS13 activity, which may be explained by underlying endothelial cell injury [27]. Thus low ADAMTS13 levels are correlated with higher risk of TMA and poor outcome in COVID-19 patients. During normal pregnancy ADAMTS13 is being produced also by the placenta and reaches higher concentration than in non-pregnant individuals [28], with the highest concentration in the first trimester, which gradually declines in the second, third trimester and at term pregnancy [29]. This decline is consistent with reduced placental expression of ADAMTS13 throughout the pregnancy that correlates with decreased placental perfusion [30]. What more the decreased (serum and placental) ADAMTS13 levels were found during ischaemic conditions and in severe PE [30,31]. Figure 1 depicts the processes described above.

#### Effects of SARS-CoV-2 infection on the placenta

We suspect that COVID-19 may either mimic initiate or act synergistic with an underlying microvascular endothelial injury and endothelitis fuelling vasoconstriction and ischaemia [11]. Together with pregnancy induced hypercoagulability COVID-19 greatly increases thromobitic risk. All the aforementioned changes may contribute to an increased risk of negative pregnancy outcomes in patients with pre-existing PE during SARS-CoV-2 infection. The next step is to consider evidence whether SARS-CoV-2 infection during first and early second trimester of pregnancy disrupts trophoblast invasion and triggers vascular changes with PE being the final outcome (Figure 1) [32,33].

1. Due to SARS-Cov-2 affinity to ACE-2 receptor, it’s higher expression in pregnancy may cause higher risk of infection during pregnancy
2. ACE-2 is expressed in endothelial cells and when they become infected by SARS-CoV-2 it may lead to immune mediated endothelial injury
3. Endothelitis causes compliment activation and formation of C5b-9 complex causing activation of the common coagulation pathway. Neutrophils are attracted to the loci of endothelial injury, they may undergo decondensation and expansion of chromatin and release of granules to the space surrounding neutrophils – process that is known as NET formation.
4. NETs express presence of PAD4 an enzyme that may change ADAMTS13 structure reducing it’s potential to destroy von Willebrand factor multimers promoting activation of platelets.
5. Points (3) and (4) sum up to result in higher TMA risk and induction of ischaemic changes, that is, in the placenta
6. SARS-CoV-2 causes ACE-2 downregulation and may accelerate RAS dysregulation summing it up with a pre-existing potential to develop PE or initiating the cascade of changes that may develop into late-onset PE
7. Following RAS dysregulation a shift in the balance between Ang II/Ang-(1-7) occurs adding the vasoconstrictive effect, increasing blood pressure, further damaging endothelial cells and in effect mimicking or worsening vascular dysfunction in PE
8. All those aforementioned changes may be the agents behind PE, FGR or PTB

Placental expression of SARS-CoV-2 cell entry proteins (ACE2 and TMPRSS2) is highest in the first trimester, with declining levels throughout the progress of the pregnancy. What is notable those levels are unchanged in pregnancies complicated by PTB (iatrogenic or spontaneous) or in those where PE occurred [34]. The implication seems clear: placenta during the first trimester is more vulnerable to SARS-CoV-2 infection, this is supported by the observations of negative outcomes from SARS and MERS epidemics [35], as well as proven placental presence of SARS-CoV-2 regardless whether the infection occurred in second or third trimester [36,37]. This may be the key factor in developing inflammation and oxidative stress in the placental tissue, which is associated with PE [38].

In an *in-silico* study proteins that interact with SARS-CoV-2 were found in placental tissue (namely TLE3 and LOX). Levels of both of them were increased in early as well as at term pregnancies [39]. TLE3 and LOX play a role in placentation and arterial remodelling, and their downregulation (in transgenic TLE3-deficient mice) has been associated with in utero demise, as well as abnormal placental development, major changes in maternal vasculature and spiral artery remodelling [40]. This finding is concordant with histological findings in placental tissue collected from pregnant COVID-19 patients where decidual arteriopathy including atherosis, fibrinoid necrosis and mural hypertrophy of membrane arterioles was reported [41]. Features mentioned above are parts of maternal vascular malperfusion (MVM), which is found in pregnancies complicated by FGR, preterm birth and oligohydramnios [42,43]. Prior to COVID-19 pandemic MVM was most commonly diagnosed in cases of PE [44,45].

#### Controversies concerning PE incidence in COVID-19 patients

Even though the pathological pathways leading to PE in COVID-19 patients show logical continuity there are discrepancies between studies reporting it’s incidence. In a case-control study performed by Guido et al. [46] on a group of 203 COVID-19 (+) and 197 COVID-19 (–) pregnant women has shown that PE was diagnosed in 10.3% of COVID-19 (+) and 13.1% COVID-19 (–) patients. Although the group reported no statistical significance to these findings. What weighs importance is a highlight to this study pointing to other comorbidities such as chronic hypertension (33.4%) and obesity (60.0%) in women with SARS-CoV-2 infection were associated with PE. In PE negative group it was respectively 5.5% and 32.8% with results being statistically significant. Another study by Tran et al. [47] also reported no significance in prevalence of PE in pregnant patients. The authors report 3.2% incidence of PE out of 93 SARS-CoV-2 infected women and in the group of 186 SARS-CoV-2 negative women 2.2% were diagnosed with PE. Marchand et al. [48] performed a systemic review of 111 studies comprised of 42754 pregnant women with SARS-CoV-2 infection. They have found a 7% rate of PE and an OR 1.6 in infected women. However when considering the quality of the studies and a heterogenous testing regime and patient group they concluded that these findings where not significant.

In a study by a group from Mexico [49] an increase in PE rate in third trimester patients with SARS-CoV-2 infection (18%) when compared to negative patients (9%) with OR 2.2. Further support is found in the INTERCOVID cohort study performed by Villar et al. [50]. The authors report higher incidence of pregnancy induced hypertension (RR 1.46) and PE/eclampsia/HELLP (RR 1.76) in COVID-19 patients when compared to COVID-19 negative women. Difference in findings were addressed in a systemic review by Tossetta et al. [51]. The group pointed to the fact that COVID-19 may cause a PE-like syndrome thus we may hypothesize this may a be a factor that caused higher incidence rates in some studies. Moreover most studies do not discriminate different severity grades of COVID-19.

A possible explanation to these discrepancies and an insight to the effects and importance of ACE2 regulation in the placental response against SARS-CoV-2 was delivered by Taglauer et al. [52] comparing ACE2 placental expression in chorionic villi and maternal serum ACE2 in COVID-19 patients. Transmembrane ACE2 is comprised of an ectodomain to which SARS-CoV-2 may bind [53,54]. Although in response to viral or bacterial infection this ectodomain may undergo shedding forming a soluble form of ACE2 still enzymatically active and able to bind viral particles [52]. As such this soluble ACE2 may act as a decoy receptor to inert SARS-CoV-2 and is being trialled as a therapy agent [54]. Still alternative routes of SARS-CoV-2 cellular entry may be enabled by this soluble ACE2 [52]. The main finding of the aforementioned study is the lower placental expression of ACE2 in third trimester SARS-CoV-2 infection in comparison of second trimester infection and healthy patients. The decreased ACE2 expression was lower in the same samples were serum ACE2 was increased. What was also proven was the fact that ACE2 was not expressed in the endothelium of foetal blood vessels of the placenta. This may provide an answer for the different results found in the studies and support the hypothesis that PE diagnosed in COVID-19 third trimester patients is in fact a PE-like syndrome. As well as supports the findings of low (0.5%−2.5%) vertical transmission rates of SARS-CoV-2 to the foetus [37].

### COVID-19 immune response and risk of preterm birth

#### Immune shift in pregnancy may have an effect on the course of viral infection

Severe COVID-19 pathogenesis is caused by dysregulation of inflammatory cascade; however, this process may become attenuated in pregnant patients thus ensuring more bening or even asymptomatic course of the disease. The response of the pregnant women to SARS-CoV-2 infection may be partially dependant on the age of the gestation at which it occurs. During the first trimester a pro-inflammatory state that benefits implantation of the embryo is observed, followed by an anti-inflammatory suppression, promoting foetal growth, in second trimester, to finally reach the third trimester with yet another pro-inflammatory shift promoting cervical maturation and labour onset [55]. It is important to note that throughout the duration of pregnancy the immune shift is a very subtle process not a simple immunosuppression, as it was proven that deletion of immune cells in the implantation site leads to early pregnancy loss [55].

#### COVID-19 effect on pro-inflammatory factors

**Figure 2.**
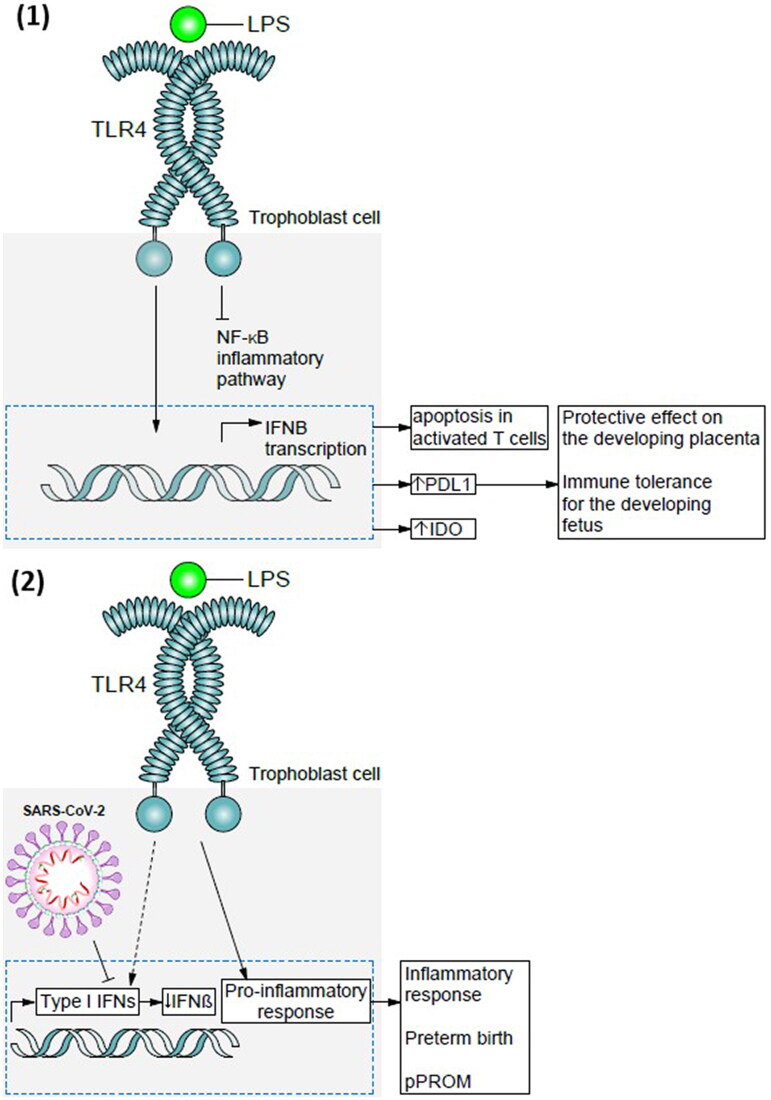
The double-hit hypothesis.

There is broad evidence supporting the fact that viral infection in the gravid mother can have a negative effect on the pregnancy, cause birth defects or pregnancy loss [56]. MERS-CoV and SARS-CoV epidemics proved that spontaneous abortion, premature birth, and FGR may be an effect of the viral infection. But it must be stated that vertical transmission of SARS-CoV-2 infection is extremely rare [35,57]. The events that trigger ARDS in COVID-19 are elevated serum levels of proinflammatory cytokines such as IL-1β, IL-2, IL-6, IL-7, IL-17 and tumour necrosis factor α (TNF-α), interferon gamma (INF-γ) [58,59]. The rise of those cytokine concentration is due to interstitial pneumonia caused by SARS-CoV-2 and is particularly high in severe course of COVID-19 [60]. The resulting cytokine storm may be the agent behind macrophage activation syndrome (MAS), and the hyperresponsive immune system is initially confined in the lung parenchyma but extends gradually onto lymphoid tissue, and through pulmonary blood vessels onto other organs [60]. Evidence for inflammation occurring in the placental tissue in SARS-CoV-2 infected patients has been delivered by Juttukonda et al. [37]. It has been observed that levels of macrophage and NK cells were higher in third trimester infection in comparison to second trimester infection and negative control group. In comparison lymphocyte T levels were higher only in third trimester infection. Cytokine expression in the placenta is also correlated with increased macrophage levels, as third trimester cytokine levels were found to be higher and second trimester infection was correlated with downregulation of these cytokine levels over time. This may point to an adaptive immune response in third trimester SARS-CoV-2 infection, but also that earlier infections during pregnancy show signs of resolving immune response at term [37]. Further evidence for resolving infection was found observing first trimester SARS-CoV-2 infection and vaccinated population in comparison to SARS-CoV-2 negative group. Levels of decidual macrophages, NK cells, T cells and cytokines were matching in all these subgroups (Figure 2) [61].

1. **In absence of viral infection maternal microbiota is responsible for baseline type I IFN production.** TLR4 on the surface of trophoblast cell respond to LPS and as an effect of TLR4 and LPS connection produce type I IFN and NF-κB inflammatory pathway is inhibited. Thus production of IFNβ begins—an agent able to modulate inflammatory process. This causes apoptosis in activated T cells and increase in production of PDL1 and IDO all of which have a protective effect for the placental tissue. This promotes immune tolerance for the developing foetus.
2. **In cells** **infected** **with SARS-CoV-2 the following scenario may occur.** Presence of a viral infection modification of TLR4 pathways occurs which promotes a pro-inflammatory response. Production of IFNβ is attenuated and decreased IFNβ levels may cause disability of immune cells to control and respond to commensal microbiota. This causes further inflammatory cascade to take place and increases the risk of pPROM and PTB.

#### COVID-19 and the ‘double-hit hypothesis’

One of the potential pathways of inducing PTB in COVID-19 patients is a ‘double-hit hypothesis’ of bacterial and viral co-infection [55]. The basis of the hypothesis is presence of maternal microbiota in the materno-foetal interface responsible for a baseline IFNα and IFNβ (type I interferons) production which is modulating maternal immune system and has a potential to induce immune tolerance for the developing foetus [62]. It is proven that trophoblast cells express TLRs and NLRs in response to bacterial lipopolysaccharides (LPS), as an effect of TLR4 and LPS connection trophoblast cells start the production of type I IFN occurs rather than nuclear factor (NF-κB) inflammatory complex [55,63]. IFNβ has been proven to be expressed in mammalian placental tissue, it possess the ability to: induce an antimicrobial state, modulate innate immune responses, activate adaptive immune system [64–66]. The point of effect of IFNβ is the TLR signalling pathway, through which it is able to modulate inflammatory process [67,68]. IFNα and IFNβ are capable of inducing apoptosis in activated T cells and in the same time increase IL-10, programmed cell death 1 ligand 1 (PDL1) and indoleamine 2,3 dioxygenase (IDO) all of which have a protective effect for the placental tissue [69–71]. The ‘double-hit hypothesis’ explains how a viral infection can attenuate IFNβ expression stopping the placental antiviral activity (Figure 2) [55]. Presence of a viral infection modifies TLR4 pathways present as a reaction to pregnant women’s microbiota, and promotes a pro-inflammatory response [62]. As the conclusion may be elucidated that IFNβ promotes immune cell receptivity and tolerance for the foetal antigens at the materno-foetal border, viral infection may result in decreased IFNβ levels causing disability of immune cells to control and respond to commensal microbiota [55,72]. It was reported by Cardenas et al. in an animal study, that induction of viral infection and stimulation with low doses of LPS resulted in reduction of IFNβ production, cytokine storm (with high levels of C-X-C motif chemokine ligand 10 (CXCL10), granulocyte-colony stimulating factor (G-CSF), TNF, IL 8—all associated with labour induction) and finally preterm birth [73–75]. These cytokine shifts were not observed in cases were LPS was administered without viral infection, nor when viral infection was induced but LPS was not administered [76]. This leads to the conclusion that a viral infection such as SARS-CoV-2 may infect trophoblast cells and change their response from immune tolerance to pro-inflammatory promoting preterm birth.

#### Effect of pregnancy associated immune shift on the course of COVID-19

When considering the response of the pregnant women with COVID-19 it is essential to take into account the Th1/Th2 immune shift. The physiological attenuation of the proinflammatory Th1 related cytokines, lasting until third trimester [55], can be a protective factor form extensive pulmonary damage and ARDS, as it was reported that IL-1β, IL-6, TNF-α are a good marker of cytokine response (and cytokine storm), with their lower serum levels associated with higher survival rates [77]. On the other hand Th2 related cytokines (IL-4, IL-10, IL-13 and transforming growth factor beta (TGF-β)) have an anti-inflammatory potential. In pregnant COVID-19 patients the shift from Th1 to Th2 related response [78] has the ability to decelerate a dysregulated immune response not allowing the cytokine storm to occur [60]. Upon third trimester the Th1 response becomes augmented and the response on SARS-CoV-2 infection has the potential to become dysregulated, on the same level as in a non-pregnant patients but with all the drawbacks of the changes in the respiratory and cardiovascular system that occur in pregnant women. This may have an effect on the rate of PTB, as it was reported that PTB and caesarean delivery (CD) is more common in SARS and MERS infection as well as in COVID-19 [32,50,79], according to Adhikari et al. [80] the rate of PTB is the same in SARS-CoV-2 positive as in SARS-CoV-2 negative group (RR 1.02). On the other hand Allotey et al. [81] report in their ‘living review and meta-analysis’ higher rates of PTB In SARS-CoV-2 positive women (OR 1.47). What question is still present is the cause of PTB as most of COVID-19 patients are diagnosed in third trimester, and in those patients the decision of ending the pregnancy *via* CD may be dictated by deteriorating condition of the mother and ongoing pneumonia. In aforementioned studies [37,52,61] presenting evidence of immune response in the placental tissue of patients with third trimester infection and resolving inflammation at term when infection occurred in second trimester a controversy remains. The studied group only had 1 patient that required hospital treatment for COVID-19 and all of the samples that were collected for the study were taken at term delivery. This points out to a pre-selected group of women that did not develop PTB nor pRPOM as well as there was no need for obstetric intervention and earlier delivery. From the previous chapter we reviewed MVM and changes associated with endothelitis in the placenta. These changes may have the potential to induce spontaneous PTB with pPROM (premature preterm rupture of membranes) being the clinical manifestation [34]. The data published so far shows scarce percentage of pregnant patients that were diagnosed in first and only slightly higher number of patients in second trimester. We assume that most of these patients were suffering from symptomatic COVID-19 with moderate or severe course. We lack data concerning asymptomatic COVID-19 patients in the first and second trimester of pregnancy, and the outcomes of pregnancy in this population. Whether the rates of PTB or PE in this group is higher remains to be proven in future studies.

## Conclusions and further study directions

### Our conclusions from this review are as follows

- PTB may not be iatrogenic due to maternal complications in the third trimester (associated with ARDS in the mother), but the cascade that will eventually trigger labour may be initiated in patients that suffered SARS-CoV-2 infection in first or second trimester (whether asymptomatic or symptomatic).
- PE may be a complication occurring in patients that had undergone COVID-19. Changes that occur in the decidua and early placenta during SARS-CoV-2 infection in the first and second trimester, may carry the risk of developing PE, HELLP, hypertension or FGR in the third trimester. This still needs to be differentiated from PE-like syndrome
- The experiences we earn from COVID-19 in pregnant patients may be in future extrapolated onto other viral epidemics. A fact that might help decrease number of severe cases or complications that medical personnel may encounter in severe viral infection in pregnant population.

## Data Availability

Data sharing is not applicable to this article as no new data were created or analysed in this study.
